# Veterinary homeopathy: Systematic review of medical conditions studied by randomised trials controlled by other than placebo

**DOI:** 10.1186/s12917-015-0542-2

**Published:** 2015-09-15

**Authors:** Robert T Mathie, Jürgen Clausen

**Affiliations:** British Homeopathic Association, Hahnemann House, 29 Park Street West, Luton, LU1 3BE UK; Karl und Veronica Carstens-Stiftung, Am Deimelsberg 36, D-45276 Essen, Germany

**Keywords:** Veterinary homeopathy, Randomised controlled trials, Systematic review

## Abstract

**Background:**

No systematic review has previously been carried out on randomised controlled trials (RCTs) of veterinary homeopathy in which the control group was an intervention other than placebo (OTP). For eligible peer-reviewed RCTs, the objectives of this study were to assess the risk of bias (RoB) and to quantify the effect size of homeopathic intervention compared with an active comparator or with no treatment.

**Methods:**

Our systematic review approach complied fully with the *PRISMA* 2009 Checklist. Cochrane methods were applied to assess RoB and to derive effect size using standard meta-analysis methods. Based on a thorough and systematic literature search, the following key attributes of the published research were distinguished: individualised homeopathy (n = 1 RCT)/non-individualised homeopathy (n = 19); treatment (n = 14)/prophylaxis (n = 6); active controls (n = 18)/untreated controls (n = 2). The trials were highly diverse, representing 12 different medical conditions in 6 different species.

**Results:**

No trial had sufficiently low RoB to be judged as reliable evidence: 16 of the 20 RCTs had high RoB; the remaining four had uncertain RoB in several domains of assessment. For three trials with uncertain RoB and without overt vested interest, it was inconclusive whether homeopathy combined with conventional intervention was more or was less effective than conventional intervention alone for modulation of immune response in calves, or in the prophylaxis of cattle tick or of diarrhoea in piglets.

**Conclusion:**

Due to the poor reliability of their data, OTP-controlled trials do not currently provide useful insight into the effectiveness of homeopathy in animals.

**Electronic supplementary material:**

The online version of this article (doi:10.1186/s12917-015-0542-2) contains supplementary material, which is available to authorized users.

## Background

Our group’s systematic analysis of the published literature of randomised controlled trials (RCTs) in veterinary homeopathy identified 38 peer-reviewed papers that we regarded as potentially eligible for detailed review [1]. As emphasised in that article, no systematic review of this research evidence had ever previously been carried out.

We have recently reported our review findings for 18 placebo-controlled trials of veterinary homeopathy [2, 3], in which there was indecisive evidence whether the use of homeopathy in animals is distinguishable from placebos. The present paper reports our results from a similarly detailed appraisal and analysis of the remaining papers, each of which reported an RCT where the control group was an intervention other than placebo (OTP).

Our approach here reflects our original literature analysis [1] and so we continue to distinguish peer-reviewed from non-peer-reviewed articles, individualised from non-individualised homeopathy, and treatment from prophylaxis. We thus report our findings from the appraisal of peer-reviewed, OTP-controlled trials of veterinary homeopathy (individualised or non-individualised, treatment or prophylaxis).

Our objective firstly was to assess the study quality (risk of bias) of each eligible RCT [4], together with the direction and statistical significance of treatment or prophylactic effect. For suitable groupings of RCTs (per species-specific medical condition; per type of control group), we aimed then to determine pooled summary statistics by meta-analysis methods and to examine their sensitivity to study quality. In both objectives, our emphasis was focused on trials that satisfied our criteria for reliable evidence [2, 3].

## Methods

Methods comply with the *PRISMA (Preferred Reporting Items for Systematic Reviews and Meta-Analyses)* 2009 Checklist (see Additional file 1) [5]. They are compatible with our previously published papers in this series of veterinary reviews [1–3], and with our protocol-based systematic reviews and meta-analyses of homeopathy RCTs in human medicine [6, 7].

Matters connected with study eligibility, research design categories and the literature search strategy were described in detail in our earlier paper [1]. Only brief descriptions are therefore given here, with additional information that is specific to the methods used for the present paper.

### Identifying papers for full data extraction

Each of the following electronic databases was searched up to and including March 2011 [1], with a follow-up search of the same databases up to the end of 2013: *AMED*, *CINAHL*, *CENTRAL*, *Embase*, *HomVetCR*, *LILACS*, *PubMed*, *Science Citation Index*, *Scopus*. There were no language restrictions. During the peer-review process for the current paper, we became aware that relevant additional literature might be contained in *CAB Abstracts* [8], which we therefore searched up to and including May 2015, and with the intention to update our review and analysis if significant numbers of eligible RCTs were revealed.

In our original literature search [1], 20 records of trials were identified as satisfying the key acceptance criteria for the present systematic review: substantive report of clinical treatment or prophylaxis trial for any medical condition or species in veterinary homeopathic medicine, randomised, controlled by OTP, and published in a peer-reviewed journal. One of us screened and categorised each of these potentially relevant papers (plus those identified in our follow-up search – see *Results*) to assess their eligibility for full data extraction. The other independently appraised these decisions; any differences of opinion were resolved by consensus discussion.

#### Exclusion criteria prior to full data extraction

- Research using radionically prepared ‘homeopathic’ medicines.
- The tested intervention is homeopathy combined with other (complementary or conventional) medicine or therapy.

### Data extraction and management

The authors of eligible RCT papers were not approached for clarification on unclear or missing facets of their methods or results [4], though original authors’ cross-reference to their previously published study methods were taken into account as appropriate [2]. For each of two assessors working independently, relevant data were extracted and then recorded using a standardised data collection format (Microsoft *Excel*).

No paper reported more than one RCT. For a paper reporting an RCT that involved >2 groups of subjects, we typically pursued data extraction on *only one pair of groups*: trials that included an OTP as well as a placebo control group had been scrutinised previously [2, 3] and were not reappraised for the current study. For studies that comprised >1 homeopathy group, the total sample size reflects the total numbers of subjects in the homeopathy groups *combined* [9]. This was the approach in all relevant cases, i.e.: where the same homeopathic medicine was used, and with the same timing of administration but in different potency; where the same homeopathic medicine and potency was used, but with different timing of administration; where a different homeopathic medicine was used.

### Study appraisal

#### Assessment of risk of bias

Using the standard criteria defined by Cochrane [4], extraction of information from each paper enabled us to answer the question, ‘Is the study free from risk of bias?’: ‘Yes’; ‘Unclear’; ‘No’. The two assessors’ independent judgments were mutually scrutinised and compared, with discrepancies between them resolved by consensus discussion. This approach applied to each of seven assessment domains: I, the method used to generate the random sequence; II, the method of allocation concealment used to implement the random sequence; IIIA, the blinding of trial personnel, including animal owner as appropriate; IIIB, the blinding of outcome assessors; IV, whether all the randomised patients are accounted for in the analysis; V, whether there is evidence of selective outcome reporting; VI, whether there is evidence of other bias, such as extreme data imbalance at baseline.

For domain IV, a trial was automatically regarded as no better than ‘unclear’ if there was greater than 20 % participant attrition rate, irrespective of whether intention-to-treat analysis had been carried out on the data. For domain V, we based our judgment of reported outcomes on a comparison with the details given in the same paper’s *Methods* section (original trial protocols have not been published in veterinary homeopathy). For the purposes of the current paper, we assessed domain V as ‘No’ (high risk of bias) if the main outcome data were not extractable for meta-analysis. The source of any research funding/sponsorship, or other vested interest such as personnel employment or contract, was not taken into account for risk-of-bias assessment (domain VI), but was reflected in the overarching assessment of risk of bias for each RCT.

#### Assessment per trial for risk of bias

Using the Cochrane approach [4], each trial was designated overall as follows: low risk of bias for all domains; uncertain risk of bias for one or more domains, and no evident risk of bias in any domain; high risk of bias for one or more domains. A trial with overall low risk of bias comprised reliable evidence. For a trial that did not display high risk of bias in any domain, we regarded its evidence as reliable if the study was assessed as free of bias for each of domains I, IIIA, IIIB and IV [2, 3].

### Outcome assessment and reporting

#### Identification of ‘main outcome measure’ per trial

For the purposes of risk-of-bias assessment and for assessment of treatment effect, we identified for each trial a single ‘main outcome measure’ using a refinement of the approaches adopted by Linde et al. [10] and by Shang et al. [11]. The main outcome measure of each trial was based on a hierarchical ranking order (consistent with the World Health Organization’s [12] classification system for levels of functioning linked to health condition), and as previously described [2]. This approach ensured that the most clinically important outcome was selected per trial, and also avoided the problem of outcome multiplicity [13].

Unless otherwise indicated, the single end-point (as determined from the start of the intervention) associated with the designated ‘main outcome measure’ was taken as the last follow-up at which data were reported for that outcome [2].

### Analysis of outcomes

#### Summary effect measures for ‘main outcome’

For each eligible trial, the effect size was taken as the difference between the homeopathy group and the control group at the designated end-point of the trial, as follows [2]:

- For a dichotomous measure: odds ratio (OR), with 95 % confidence interval (CI);
- For a continuous measure: standardised mean difference (SMD), with 95 % CI.

If the original paper did not provide adequate information on the designated main outcome measure to enable data extraction, that trial’s outcome was classified as ‘not estimable’ and a further potentially estimable outcome was not sought.

Under the separate group headings of *individualised homeopathy* and *non-individualised homeopathy*, and for the categories below in which there was >1 RCT of a given study design that had extractable data, we aimed to determine pooled summary statistics for:

- Disease-specific treatment effects per species;
- Disease-specific prophylactic effects per species.

All calculations and analyses were performed using *Review Manager 5.2* (Cochrane). Given our anticipation of heterogeneous data for intervention effects, the random-effects (rather than fixed-effects) model was planned for all meta-analyses [14].

#### Reflecting study quality overall

Our main analyses used only the data extracted from trials without evident high risk of bias. It was intended that our primary conclusions would be based solely on trials with reliable evidence and without overt vested interest (not funded, directly or indirectly, by a homeopathic pharmacy).

### Direction of effect of treatment/prophylaxis per trial

Care was taken faithfully to represent the correct direction of change per trial: e.g. an effect favouring homeopathic intervention was *higher* rate of recovery but *lower* faecal egg count. The arithmetic content of the OR, or the sign of the SMD, was adjusted appropriately if so indicated.

We regarded each RCT as a *superiority trial* [15] unless the original paper stated explicitly in its *Methods* section that the study was designed as an equivalence or non-inferiority trial [16, 17], with corresponding power calculation. For each of three study designs (and regarding each design as a superiority trial), we adopted the following rationale for interpretation of the three possible statistical findings:

Statistical finding (i): *P* ≤ 0.05: Direction of effect toward homeopathy

- *Study design: active control.* Homeopathy is more effective than a conventional intervention;
- *Study design: [homeopathy plus active control]* versus *active control (‘[A + B]* versus *B’).* Homeopathy combined with conventional intervention is more effective than conventional intervention alone;
- *Study design: no-treatment control.* Homeopathy is more effective than no intervention.

Statistical finding (ii): *P* ≤ 0.05: Direction of effect toward control

- *Study design: active control.* Homeopathy is less effective than a conventional intervention;
- *Study design: [homeopathy plus active control]* versus *active control (‘[A + B]* versus *B’).* Homeopathy combined with conventional intervention is less effective than conventional intervention alone;
- *Study design: no-treatment control.* Homeopathy is ineffective.

Statistical finding (iii): *P* > 0.05: Direction of effect toward either homeopathy or control

- *Study design: active control.* Inconclusive whether homeopathy is more or is less effective than a conventional intervention [18, 19];
- *Study design: [homeopathy plus active control]* versus *active control (‘[A + B]* versus *B’).* Inconclusive whether homeopathy combined with conventional intervention is more or is less effective than conventional intervention alone;
- *Study design: no-treatment control.* Inconclusive whether homeopathy is more or less effective than no treatment.

## Results

### Demographic details

Additional file 2 illustrates the *PRISMA* flowchart, which follows on from, and suitably updates [20], that in our earlier paper [1]; our current focus is on OTP-controlled trials only. Two OTP-controlled trials were rejected from the current review due to their focus on homeopathy combined with another intervention: A29, Dreissman 2010; A30, Lepple 1984. A further two were excluded due to their focus on neither treatment nor prophylaxis: A33, Sharma 1987; A37, Trehan 1994. Four additional eligible records were identified in our follow-up search, and included in analysis. Our subsequent search of *CAB Abstracts*, conducted during peer review of the current paper, revealed just one potentially eligible trial [21], which we did not consider further.

Table 1 details each of the 20 studies eligible for full systematic review: (i) individualised homeopathy/treatment (n = 1); (ii) non-individualised homeopathy/treatment (n = 13); (iii) non-individualised homeopathy/prophylaxis (n = 6). Data per trial include: nature of the homeopathic intervention; study setting; the RCT’s source of funding. Our definitions of ‘treatment’ and ‘prophylaxis’ were as previously described [1]. All RCTs were identified as superiority trials, despite the fact that in some cases the original authors applied the terms ‘equivalence trial’ or ‘non-inferiority trial’ *post-hoc* in *Results* or in *Discussion* (A15, Faulstich 2006; A39, Braun, 2011).

**Table 1:** Demographic details of 20 OTP-controlled RCTs in veterinary homeopathy

**i: Individualised/Treatment**											
**Condition**	**Species**	**Ref.**	**First author**	**Year**	**Design**	**Control**	**Homeopathic medicine**	Dilution*	**Study setting**	**Funding**	**Free from vested interest**
Mastitis, metritis and agalactia	Pigs	A12	Schütte	1988	Active	Antibiotic	Individualised	D3-D12	University veterinary ambulance; 21 swine herds in Germany (Berlin area)	Remedies were gift of the manufacturer	No
**ii: Non-individualised/Treatment**											
**Condition**	**Species**	**Ref.**	**First author**	**Year**	**Design**	**Control**	**Homeopathic medicine**	**Dilution**	**Study setting**	**Funding**	**Free from vested interest**
Diarrhoea (neonatal)	Cattle	A42	Lohr	2012	Active	Oral electrolytes	3 complex preparations: Nux vomica; Veratrum; Engystol	Not stated	Six farms in Germany	Directly funded study (Heel Pharmacy)	No
Diarrhoea (neonatal)	Pigs	A14	Coelho	2009	Active	Antibiotic	Phosphorus/E. coli	30C	Swine farm, Brazil	The company, Farmácia Sensitiva, 'supplied' the medicines	No
Ectoparasite infestation	Cattle	A41	Catto	2013	Active	Anthelmintic	2 complex preparations of various components (mainly nosodes)	All but one components potentised to 12C and higher (30C, 200C)	Cattle farm, Brazil	None stated	Unclear
Ectoparasite infestation	Cattle	A19	Silva	2008	Active	Chemical dip	Biotherapic' of Boophilus microplus and 14 other organisms	12C	Government research institution, Brazil	None declared'. The homeopathic laboratories, Flora & Fauna Ltd., supplied the medicine	No
Foot-and-mouth disease	Cattle	A40	Lotfollahzadeh	2012	Active	Anti-inflamatory and antibiotic	Tarentula cubensis	D5	Cattle farms, Iran	Richter Pharma donated the homeopathic medicine	No
Gastrointestinal nematodes	Sheep	A21	Zacharias	2008	Active	Anthelmintic	3 remedies: Ferrum phosphoricum, Arsenicum album, Calcarea carbonica	Not stated	Government research institution, Brazil	None stated	Unclear
Lameness	Horses	A15	Faulstich	2006	Active	Hyaluronic acid	Complex of 14 homeopathically prepared ingredients	D3 - D8	Two horse clinics in Germany (Berlin area, Munich area)	Study performed by a contract research organisation	No
Mastitis	Cattle	A16	Klocke	2010	Active	Teat-sealer	8 remedies: Mercurius solubilis, Lachesis mutus, Sulfur, Calcium carbonicum, Calcium phosphoricum, Pulsatilla pratensis, Sepia, Silica	D6	Thirteen organic dairy herds, Switzerland	EC grant; Weleda 'provided the remedies'	No
Mastitis	Cattle	A20	Varshney	2005	Active	Antibiotic	Complex of 8 remedies: Healwell VT-6 (Sintex International Limited, Kalol, India), consisting of Phytolacca, Calcarea fluorica, Silica, Belladona, Bryonia, Arnica, Conium, Ipecacuanha "in equal amount"	200C: Phytolacca, Calcarea fluorica. 30c: Silica, Belladona, Bryonia, Arnica, Conium, Ipecacuanha	Dairy farm, India	Sintex International Ltd 'supplied the homeopathic formulation for clinical trial'	No
Mastitis, metritis and agalactia	Pigs	A39	Braun	2011	Active	Antibiotic	2 complex preparations. Lachesis compositum + Traumeel	Not stated	Three piglet-rearing farms in Germany	Directly funded study (Heel Pharmacy)	No
Pseudopregnancy	Dogs	A13	Beceriklisoy	2008	Active	Naloxone	Thuja occidentalis/Urtica urens	D30	University animal clinic, Turkey	None stated	Unclear
Salmonellosis	Birds	A18	Sandoval	1998	Active	Antibiotic	Baptisia tinctoria	30C	Poultry farm, Mexico	None stated	Unclear
Gastrointestinal nematodes	Sheep	A17	Rocha	2006	(A + B) vs. B	Anthelmintic only	Fator Vermes	Not stated	University establishment, Brazil	None stated	Unclear
**iii: Non-individualised/Prophylaxis**											
**Condition**	**Species**	**Ref.**	**First author**	**Year**	**Design**	**Control**	**Homeopathic medicine**	**Dilution**	**Study setting**	**Funding**	**Free from vested interest**
Ectoparasite infestation	Cattle	A34	Signoretti	2008	(A + B) vs. B	Protein supplement only	Factor C & MC ® : 16 remedies	All 12C	Government research institution, Brazil	None stated	Unclear
Diarrhoea (neonatal)	Pigs	A36	Soto	2008	(A + B) vs. B	Sucrose saline only	Complex of 4 remedies: Echinacea angustifolia, Avena sativa, Ignatia amara, and Calcarea carbonica	6C	Commercial swine herd, Brazil	None stated	Unclear
Handling stress	Cattle	A31	Reis	2006	(A + B) vs. B	Mineral salt only	Matricaria chamomilla®	12C (plus non potentised sugar and Bixa orellana [grams])	Cattle farm, Brazil	"This research was supported by the Homeopathic Laboratory Arenales Flora & Fauna Ltd."	No
Immune response to rabies vaccination	Cattle	A32	Reis	2008	(A + B) vs. B	Mineral salt only	Matricaria chamomilla®	12C	Cattle farm, Brazil	The homeopathic laboratories, Flora & Fauna Ltd., are thanked by the author non-specifically	Unclear
Infertility	Cattle	A35	Sommer	1972	Un-treated	Untreated	Complex of 5 remedies	D3-D5	Two cattle farms, University of Hohenheim (Germany)	None stated	Unclear
Infertility	Cattle	A38	Williamson	1991	Un-treated	Untreated	Sepia	200C	Dairy herd, Scotland	British Cattle Veterinary Association. The pharmacy, Ainsworth's, are thanked by the authors non-specifically	Unclear

*Note on homeopathic dilutions: The number refers to the number of successive serial dilutions to which the starting material has been subjected. The letter refers to the scale on which the dilution has been carried out: the letter D denotes the decimal method of dilution (that is, one part of liquid is added to nine parts of purified water, ethanol, glycerol or lactose); the letter C indicates the centesimal method (one part added to 99 parts of diluent). In homeopathic dilutions above 12C/D24 (10^−24^ molar) – beyond Avogadro’s constant, 6.02 x 10^23^ mol^−1^ – there are, in theory, no material traces of the original substance; such dilutions are known as ‘ultra-molecular’

Extreme diversity characterised the studies as regards species, medical condition, homeopathic medicine, and funding source. In the 20 eligible studies, 6 different species are represented: birds (n = 1); cattle (n = 11); dogs (n = 1); horses (n = 1); pigs (n = 4); sheep (n = 2). Twelve different conditions are represented. None of the trials had a clearly unbiased funding source; we identified overt vested interest in 10 of the 20 trials.

Table 2 includes: sample sizes; designated ‘main outcome measure’; type of data (whether dichotomous or continuous); study endpoint. Diversity was again apparent, with large variation in sample sizes, main outcome, and timing of the study endpoint.

**Table 2:** Sample sizes and outcomes for 20 OTP-controlled RCTs

**i: Individualised/Treatment**															
**Ref.**	**First author**	**Year**	**N start (hom.)**	**N start (cont.)**	**N start (tot.)**	**N end (hom.)**	**N end (cont.)**	**N end (tot.)**	**% Attrition**	**Designated 'main outcome'**	**Type of data**	**Direction of change favouring homeopathy**	**Problem with designated 'main outcome measure'**	**End-point**	**Notes**
A12	Schütte	1988	33	31	64	33	31	64	0.0	Piglet mortality	Dichot.	Lower	Yes: Gives percentage of dead piglets (events) but not number of piglets born (total)	28 d	N = No. of sows treated
**ii: Non-individualised/Treatment**															
**Ref.**	**First author**	**Year**	**N start (hom.)**	**N start (cont.)**	**N start (tot.)**	**N end (hom.)**	**N end (cont.)**	**N end (tot.)**	**% Attrition**	**Designated 'main outcome'**	**Type of data**	**Direction of change favouring homeopathy**	**Problem with designated 'main outcome measure'**	**End-point**	**Notes**
A42	Lohr	2013	53	56	109	46	54	100	8.3	Cure rate (Sum-score (physical condition))	Dichot.	Higher	Only 46 (Hom) and 54 (Cont) animals regularly completed the study, and are therefore the data for our calculations. The authors themselves calculated cure rate based on 50 and 56 animals respectively.	Up to 4 d	
A14	Coelho	2009	35	11	46	35	9	44	4.3	Proportion of animals without diarrhoea at the end of treatment	Dichot.	Higher	No	12 d	
A41	Catto	2013	72	36	108	72	36	108	0.0	Faecal egg count	Contin.	Lower	Yes: Unclear sample sizes – see Notes	Second year	The numbers of animals in each group based on the assumption that each group comprised 3 paddocks (12 animals in each). No information on dropouts.
A19	Silva	2008	9	9	18	?9	?9	?18	Unclear	Mean number of engorged ticks per annum	Contin.	Lower	Yes: SD not stated; N at endpoint not stated	1 yr	
A40	Lotfollahzadeh	2012	52	23	75	50	15	65	13.3	Rectal temperature	Contin.	Lower		14 d	
A21	Zacharias	2008	7	7	14	?7	?7	?14	Unclear	Faecal egg count	Contin.	Lower	No. Numbers for analysis interpolated from Figure 1A. N not stated for endpoint, but assumed to be N = 7.	68 d	Disparity between data (mean eggs per gram) for conv med group given in text (1,483) and by inspection of Fig 1 (1,300 approx.)
A15	Faulstich	2006	24	22	46	22	19	41	10.9	Treatment success (overall effectiveness)	Dichot.	Higher	No : Numbers for analysis from percentage data in Table 9	21 d	Control really active?
A16	Klocke	2010	32	36	68	32	36	68	0.0	Absence of clinical mastitis infection	Dichot.	Higher	No: Data derived from text of Results [line 6])	First 100 days post-calving	
A20	Varshney	2005	67	96	163	67	96	163	0.0	Quarter cure-rate (non-fibrosed)	Dichot.	Higher	No : Numbers for analysis from percentage data in Table 2	Up to 28 d	N = quarters, not animals
A39	Braun	2011	30	34	64	28	32	60	6.3	Cure rate (MMA-sum score)	Dichot.	Higher		Up to 4 d (H) or 3 d (C)	
A13	Beceriklisoy	2008	30	8	38	30	8	38	0.0	Treatment 'success' (recovery rate)	Dichot.	Higher	No	Up to 20 d	
A18	Sandoval	1998	200	200	400	200	200	400	0.0	Cumulative total mortality (1st quality broilers)	Contin.	Lower	Yes: Data are presented as accumulated mortality – not amenable to meta-analysis	49 d	N = 1st quality broilers
A17	Rocha	2006	10	10	20	10	10	20	0.0	Proportion of animals not requiring anti-helminthic treatment	Dichot.	Higher	No: Data derived from text of Results (second paragraph)	4 mo (first phase)	
**iii: Non-individualised/Prophylaxis**															
**Ref.**	**First author**	**Year**	**N start (hom.)**	**N start (cont.)**	**N start (tot.)**	**N end (hom.)**	**N end (cont.)**	**N end (tot.)**	**% Attrition**	**Designated 'main outcome'**	**Type of data**	**Direction of change favouring homeopathy**	**Problem with designated 'main outcome measure'**	**End-point**	**Notes**
A34	Signoretti	2008	8	8	16	?8	?8	?16	Unclear	Faecal egg count per gram	Contin.	Lower	No. But assumes zero attrition rate. Data from Table 3.	30 d	
A36	Soto	2008	24	24	48	24	24	48	0.0	Number of animal-days with diarrhoea	Dichot.	Lower	No. But relate 7-day cumulated data to total possible number of animal-days per group	7d period	Hom plus sucrose saline vs. sucrose saline only
A31	Reis	2006	30	30	60	30	30	60	0.0	Serum cortisol	Contin.	Lower	No. Assumes zero attrition rate based on legend for Figure 1.	60 d	Hom plus mineral salt vs. mineral salt only
A32	Reis	2008	15	15	30	15	?15	?30	Unclear	Rabies-neutralizing antibody titer	Contin.	Higher	No. But assumes zero attrition rate.	60 d after vaccination	Compares Group FEV2 (Hom + salt) with Group V2 (salt only)
A35	Sommer	1972	40	18	58	40	18	58	0.0	Number with infertility disorders	Dichot.	Lower	No. Data from table 2	Not stated	
A38	Williamson	1991	101	32	133	?100	?32	?132	Unclear	Number with peripartum disorders	Dichot.	Lower	Yes: Unclear tabulations	None	

hom. = homeopathy. cont. = control. tot = total. dichot. = dichotomous. contin. = continuous

### Risk of bias

Table 3 shows our risk-of-bias judgments for each eligible trial.

**Table 3:** Risk-of-bias assessments for 20 OTP-controlled RCTs

**i: Individualised/Treatment**													
**Ref.**	**First author**	**Domain** **I**	**Domain** **II**	**Domain** **IIIA**	**Domain** **IIIB**	**Domain** **IV**	**Domain** **V**	**Domain** **VI (excl. fund)**	**No. of domains for which criteria fulfilled**			**Risk of bias (excl. vested interest)**	**Reliable evidence**
									Y	U	N		
A12	Schütte	N	N	N	N	Y	N	U	1	1	5	High	No
**ii: Non-individualised/Treatment**													
**Ref.**	**First author**	**Domain** **I**	**Domain** **II**	**Domain** **IIIA**	**Domain** **IIIB**	**Domain** **IV**	**Domain** **V**	**Domain** **VI (excl. fund)**	**No. of domains for which criteria fulfilled**			**Risk of bias (excl. vested interest)**	**Reliable evidence**
									Y	U	N		
A42	Lohr	Y	U	N	Y	Y	N	N	3	1	3	High	No
A14	Coelho	U	U	N	U	Y	Y	N	2	3	2	High	No
A41	Catto	U	U	N	U	U	N	U	0	5	2	High	No
A19	Silva	U	U	N	U	U	N	U	0	5	2	High	No
A40	Lotfollahzadeh	U	U	N	Y	U	N	Y	2	3	2	High	No
A21	Zacharias	U	U	N	U	U	Y	U	1	5	1	High	No
A15	Faulstich	Y	U	N	Y	Y	Y	Y	5	1	1	High	No
A16	Klocke	Y	U	N	U	Y	Y	Y	4	2	1	High	No
A20	Varshney	U	U	U	U	U	Y	N	1	5	1	High	No
A39	Braun	Y	U	N	Y	Y	Y	N	4	1	2	High	No
A13	Beceriklisoy	U	U	U	U	U	Y	N	1	5	1	High	No
A18	Sandoval	U	U	U	U	U	N	U	0	6	1	High	No
A17	Rocha	N	N	U	U	Y	Y	U	2	3	2	High	No
**iii: Non-individualised/Prophylaxis**													
**Ref.**	**First author**	**Domain** **I**	**Domain** **II**	**Domain** **IIIA**	**Domain** **IIIB**	**Domain** **IV**	**Domain** **V**	**Domain** **VI (excl. fund)**	**No. of domains for which criteria fulfilled**			**Risk of bias (excl. vested interest)**	**Reliable evidence**
									Y	U	N		
A34	Signoretti	U	U	U	U	U	Y	Y	2	5	0	Uncertain	No
A36	Soto	U	U	U	U	Y	Y	U	2	5	0	Uncertain	No
A31	Reis 2006	U	U	U	U	U	Y	Y	2	5	0	Uncertain	No
A32	Reis 2008	U	U	U	U	U	Y	Y	2	5	0	Uncertain	No
A35	Sommer	U	U	N	U	Y	Y	U	2	4	1	High	No
A38	Williamson	U	U	N	U	U	N	U	0	5	2	High	No

Domains I-VI are explained in the main text. Criteria fulfilled for domains: Y = Yes; U = Unclear; N = No

Many of the papers were written to such a poor standard that risk-of-bias assessments were sometimes challenging. No trial had low risk of bias in all of the Cochrane judgmental domains. Sixteen trials had high risk of bias in one or more domains. Four trials had merely uncertain risk of bias, and the uncertainty was evident in five domains per study (A31, Reis 2006; A32, Reis 2008; A34, Signoretti 2008; A36, Soto 2008). There was therefore no trial that could be designated as reliable evidence.

**Fig. 1:**
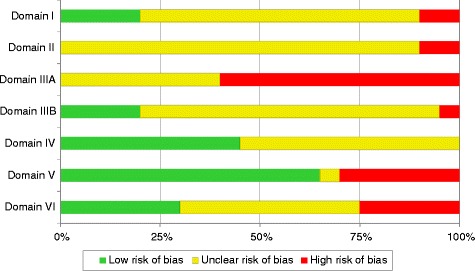
Risk-of-bias summary graph**.** Illustrates, for each assessment domain, the proportion of RCTs with low, unclear and high risk of bias

Only five of the 20 trials had adequate randomisation (domain I), and none of the trials had adequate allocation concealment (domain II) or personnel blinding (domain IIIA). See also Fig. 1 (*Risk-of-bias summary graph*).

### Analysis of outcomes

Fifteen of the 20 trials had extractable data. Due to the diversity of medical conditions, species, types of homeopathic intervention, study designs and outcome measures, as well as the very unclear quality of the evidence, it was not appropriate to carry out meta-analysis on disease-specific intervention.

The direction of effect favoured homeopathy in 11 trials (statistically significantly so in four cases) and favoured control in four trials (statistically significantly so in zero cases) – see Table 4, in which the inference from the statistical findings is given by reference to the numbered study designs in *Methods: Direction of effect of treatment/prophylaxis per trial*.

**Table 4:** Summary statistics for: (a) trials at uncertain risk of bias; (b) trials at high risk of bias

**a) UNCERTAIN RISK OF BIAS**													
**iii: Non-individualised/Prophylaxis**													
**Ref.**	**First author**	**Year**	**Condition**	**Species**	**Outcome measure**	**Hom.**	**Cont.**	**Summary effect measure**	**Effect size (95 % CI)**	**Direction of change favouring homeopathy**	**Direction of effect**	***P*** **value**	**Inference** *
A34	Signoretti	2008	Ectoparasite infestation	Cattle	Faecal egg count per gram	340 (sd, 214); N = 8	207 (sd, 131); N = 8	SMD	0.71 [−0.31, 1.73]	Lower	Cont.	0.17	[iii: b]
A36	Soto	2008	Diarrhoea (neonatal)	Pigs	Number of animal-days with diarrhoea	34/192	25/192	OR	0.70 [0.40, 1.22]	Lower	Cont.	0.20	[iii: b]
*A31*	*Reis*	*2006*	*Handling stress*	*Cattle*	*Serum cortisol*	*5.1 (sd, 1.8); N = 30*	*5.3 (sd, 1.45); N = 30*	*SMD*	*−0.12 [−0.63, 0.39]*	*Lower*	*Hom.*	*0.64*	*[iii: b]*
A32	Reis	2008	Immune response to rabies vaccination	Cattle	Rabies-neutralizing antibody titer	10.8 (sd, 9.5); N = 15	14.4 (sd, 11.1); N = 15	SMD	−0.34 [−1.06, 0.38]	Higher	Cont.	0.36	[iii: b]
**b) HIGH RISK OF BIAS**													
**i: Individualised/Treatment**													
**Ref.**	**First author**	**Year**	**Condition**	**Species**	**Outcome measure**	**Hom.**	**Cont.**	**Summary effect measure**	**Effect size (95 % CI)**	**Direction of change favouring homeopathy**	**Direction of effect**	***P*** **value**	**Inference** ^**a**^
*A12*	*Schütte*	*1988*	*Mastitis, metritis and agalactia*	*Pigs*	*None useable*	*X*	*X*	*X*	*X*	*X*	*X*	*X*	*X*
**ii: Non-individualised/Treatment**													
**Ref.**	**First author**	**Year**	**Condition**	**Species**	**Outcome measure**	**Hom.**	**Cont.**	**Summary effect measure**	**Effect size (95 % CI)**	**Direction of change favouring homeopathy**	**Direction of effect**	***P*** **value**	**Inference** *
*A42*	*Lohr*	*2013*	*Diarrhoea (neonatal)*	*Cattle*	*Cure rate (Sum-score [physical condition])*	*37/50*	*42/56*	*OR*	*0.95 [0.40, 2.28]*	*Higher*	*Cont.*	*0.91*	*[iii: a]*
*A14*	*Coelho*	*2009*	*Diarrhoea (neonatal)*	*Pigs*	*Proportion of animals without diarrhoea at the end of treatment*	*35/35*	*5/9*	*OR*	*58.09 [2.73, 1234.82]*	*Higher*	*Hom.*	*0.009*	*[i: a]*
A41	Catto	2013	Ectoparasite infestation	Cattle	None useable	X	X	X	X	X	X	X	X
*A19*	*Silva*	*2008*	*Ectoparasite infestation*	*Cattle*	*None useable*	*X*	*X*	*X*	*X*	*X*	*X*	*X*	*X*
*A40*	*Lotfollahzadeh*	*2012*	*Foot-and-mouth disease*	*Cattle*	*Rectal temperature*	*38.6 (sd, 0.2); N = 50*	*39.0 (sd, 0.1); N = 15*	*SMD*	*−2.16 [−2.86, −1.47]*	*Lower*	*Hom.*	*<0.001*	*[i: a]*
A21	Zacharias	2008	Gastrointestinal nematodes	Sheep	Faecal egg count	954 (sd, 1077); N = 7	1308 (sd, 1108); N = 7	SMD	−0.30 [−1.36, 0.75]	Lower	Hom.	0.57	[iii: a]
*A15*	*Faulstich*	*2006*	*Lameness*	*Horses*	*Treatment success (overall effectiveness)*	*18/22*	*14/19*	*OR*	*1.61 [0.36, 7.12]*	*Higher*	*Hom.*	*0.53*	*[iii: a]*
*A16*	*Klocke*	*2010*	*Mastitis*	*Cattle*	*Absence of clinical mastitis infection*	*29/32*	*32/36*	*OR*	*1.21 [0.25, 5.86]*	*Higher*	*Hom.*	*0.81*	*[iii: a]*
*A20*	*Varshney*	*2005*	*Mastitis*	*Cattle*	*Quarter cure-rate (non-fibrosed)*	*58/67*	*57/96*	*OR*	*4.41 [1.96, 9.93]*	*Higher*	*Hom.*	*<0.001*	*[i: a]*
*A39*	*Braun*	*2011*	*Mastitis, metritis and agalactia*	*Pigs*	*Cure rate (MMA-sum score)*	*20/28*	*21/32*	*OR*	*1.31 [0.44, 3.92]*	*Higher*	*Hom.*	*0.63*	*[iii: a]*
A13	Beceriklisoy	2008	Pseudopregnancy	Dogs	Treatment 'success' (recovery rate)	30/30	3/8	OR	95.86 [4.32, 2126.07]	Higher	Hom.	0.004	[i: a]
A18	Sandoval	1998	Salmonellosis	Birds	None useable	X	X	X	X	X	X	X	X
A17	Rocha	2006	Gastrointestinal nematodes	Sheep	Proportion of animals not requiring anti-helminthic treatment	7/10	5/10	OR	2.33 [0.37, 14.61]	Higher	Hom.	0.37	[iii: b]
**iii: Non-individualised/Prophylaxis**													
**Ref.**	**First author**	**Year**	**Condition**	**Species**	**Outcome measure**	**Hom.**	**Cont.**	**Summary effect measure**	**Effect size (95 % CI)**	**Direction of change favouring homeopathy**	**Direction of effect**	***P*** **value**	**Inference** ^**a**^
A35	Sommer	1972	Infertility	Cattle	Number with infertility disorders	13/40	9/18	OR	2.08 [0.67, 6.47]	Lower	Hom.	0.21	[iii: c]
A38	Williamson	1991	Infertility	Cattle	None useable	X	X	X	X	X	X	X	X

Hom. = homeopathy. Cont. = control. CI = confidence interval. sd = standard deviation. SMD = standardised mean difference. OR = odds ratio

Italic text indicates trials with a potential risk of bias due to funding source (see also Table 1)

^a^Inference from the statistical findings is given by reference to the numbered study designs in *Methods: Direction of effect of treatment/prophylaxis per trial*

#### Trials with uncertain risk of bias

In the absence of any trials with reliable evidence, our main attention has been forced on to the data extracted from three trials with uncertain risk of bias *and* without overt vested interest (Table 4a): A32, Reis 2008 (modulation of immune response to rabies vaccination in calves); A34, Signoretti 2008 (cattle tick); A36, Soto 2008 (diarrhoea in piglets). All three trials were in the category ‘non-individualised/prophylaxis/‘[A + B] versus B’ design. None of the three showed evidence of a difference between adjunctive homeopathy and active control alone, and so in each case it was statistically inconclusive whether homeopathy combined with conventional intervention was more or was less effective than conventional intervention alone – see Table 4a.

#### Trials at high risk of bias

Data could not be extracted from the single paper on individualised treatment. Data were extractable from 10 of the 13 relevant RCTs of non-individualised treatment; the direction of effect statistically significantly favoured homeopathy in four of the ten cases – see Table 4b. Two of these four yielded what appear to be spuriously high mean OR values (A13, Bekeriklisoy 2008: OR = 95.86; A14, Coelho 2009: OR = 58.09). Of the two RCTs of non-individualised prophylaxis (both with untreated controls), data were extractable from one (A35, Sommer 1972).

## Discussion

This comprehensive systematic review of the peer-reviewed literature has revealed no OTP-controlled RCT of veterinary homeopathy that comprised reliable evidence. Our main conclusions from this study are therefore restricted to the findings of three RCTs, whose quality was unclear and whose methodological diversity contraindicated meta-analysis. Each of these three trials is in the category of non-individualised homeopathic prophylaxis (A32, Reis 2008; A34, Signoretti 2008; A36, Soto 2008), a prescribing approach that some homeopathic practitioners might view as controversial [22]. Each of the three trials is also in the adjunctive category, and manifested statistically inconclusive findings: this result undermines the suggestion that the ‘[A + B] versus B’ study design can ‘generate only positive results’ [23].

For each of the above three trials, the uncertainty regarding risk of bias was so marked that their findings must be viewed with extreme caution. Given the lack of clarity in so many domains of assessment, this degree of concern would still apply even if we had reflected more leniently the fact that non-blinding of study personnel is a typical feature of an OTP-controlled trial. The admissible evidence in veterinary homeopathy for this study design is therefore unable to provide any compelling information about modulation of immune response in calves (A32, Reis 2008) or adjunctive prophylaxis of cattle tick (A34, Signoretti 2008) or of diarrhoea in piglets (A36, Soto 2008).

In assessing all the studies, we had reason on occasions to question the veracity of the control group as an ‘active’ or effective intervention (e.g. mineral or protein supplements, or naloxone in pseudopregnancy), and so raised doubts about the ‘other than placebo’ nature of some of the included trials. We were careful not to attribute ‘equivalence’ to superiority trials that found no statistically significant difference between homeopathy and active control [16, 19].

For OTP-controlled RCTs of non-individualised homeopathic *treatment*, there is nothing that we can reasonably conclude, given that each one of the trials was judged to be at high risk of bias. Thus, the positive inference we attributed to four trials in this category cannot be regarded as admissible evidence; the spuriously large treatment effect evidenced in two of the trials reinforces those serious concerns. It remains unknown whether non-individualised homeopathy is effective in the prophylaxis of fertility disorders in cattle. Likewise, no conclusions can be made regarding *individualised* homeopathic treatment, since the single trial in this category was of very low quality and yielded no extractable data for analysis.

Our most recent literature search, conducted during the process of the current paper’s peer-review, revealed only one further OTP-controlled trial that would potentially have been eligible for inclusion [21]. Notwithstanding normal concerns over the possibility that publication bias may have limited the number of ‘negative’ studies available, the present systematic review seems barely compromised by selection bias due to absence of relevant data.

Thus, the sum of the reliable peer-reviewed RCT evidence in veterinary homeopathy (out of 18 placebo-controlled and 20 OTP-controlled RCTs in total) comprises the two placebo-controlled trials that we previously identified [2, 3]. Evidence concerning the effective use of homeopathy in animals remains indecisive.

## Conclusions

Due to their extremely poor quality, OTP-controlled trials are incapable of providing useful additional insight into the effectiveness of homeopathic treatment or prophylaxis in animals. To clarify the matter, new and substantially improved OTP-controlled research in both individualised and non-individualised veterinary homeopathy is strongly indicated.

## Additional files

Additional file 1:
***PRISMA*** **2009 Checklist.** (DOC 64 kb) Additional file 2:
***PRISMA*** **flowchart, illustrating records of RCTs that are eligible and ineligible for inclusion in the systematic review.** * Records are numbered as per a previous paper published by the authors (Mathie et al., 2012) [1]. *Papers presented in italics are those identified in the follow-up literature search.* Excluded papers are shown in red. # RCT with continuous measure as main outcome (each unmarked trial has a dichotomous measure as the main outcome). (DOC 66 kb)

## Abbreviations

CAB: Commonwealth Agricultural Bureaux
CI: Confidence Interval
OR: Odds ratio
OTP: Other than placebo
PRISMA: Preferred reporting items for systematic reviews and meta-analyses
RCT: Randomised controlled trial
SMD: Standardised mean difference

## References

[1] Mathie RT, Hacke D, Clausen J (2012). Randomised controlled trials of veterinary homeopathy: Characterising the peer-reviewed research literature for systematic review. Homeopathy.

[2] Mathie RT, Clausen J (2014). Veterinary homeopathy: systematic review of medical conditions studied by randomised placebo-controlled trials. Vet Rec.

[3] Mathie RT, Clausen J (2015). Veterinary homeopathy: meta-analysis of randomised placebo-controlled trials. Homeopathy.

[4] Higgins JPT, Altman DG (Eds): Assessing risk of bias in included studies. In: *Cochrane Handbook for Systematic Reviews of Interventions*, Version 5.1.0. Edited by Higgins JPT, Green S (Eds). The Cochrane Collaboration; 2011: Chapter 8.

[5] Liberati A, Altman DG, Tetzlaff J, Mulrow C, Gøtzsche PC, Ioannidis JPA (2009). The PRISMA statement for reporting systematic reviews and meta-analyses of studies that evaluate health care interventions: Explanation and elaboration. J Clin Epidemiol.

[6] Mathie RT, Legg LA, Clausen J, Davidson JRT, Lloyd SM, Ford I: Systematic review and meta-analysis of randomised, placebo-controlled, trials of individualised homeopathic treatment: study protocol. 2013, Version 1.0; 25 January 2013. www.britishhomeopathic.org/wp-content/uploads/2013/05/Study_protocol_for_systematic_review.pdf (Accessed 19 April, 2013).

[7] Mathie RT, Lloyd SM, Legg LA, Clausen J, Moss S, Davidson JRT (2014). Randomised placebo-controlled trials of individualised homeopathic treatment: systematic review and meta-analysis. Syst Rev.

[8] Grindlay DJC, Brennan ML, Dean RS (2012). Searching the veterinary literature: A comparison of the coverage of veterinary journals by nine bibliographic databases. J Vet Med Educ.

[9] Higgins JPT, Deeks JJ, Altman DG (Eds): Special topics in statistics. In: *Cochrane Handbook for Systematic Reviews of Interventions*, Version 5.1.0. Edited by Higgins JPT, Green S (Eds). The Cochrane Collaboration; 2011: Chapter 16.

[10] Linde K, Clausius N, Ramirez G, Melchart D, Eitel F, Hedges LV (1997). Are the clinical effects of homeopathy placebo effects? A meta-analysis of placebo-controlled trials. Lancet.

[11] Shang A, Huwiler-Müntener K, Nartey L, Jüni P, Dörig S, Sterne JAC (2005). Are the clinical effects of homoeopathy placebo effects? Comparative study of placebo-controlled trials of homoeopathy and allopathy. Lancet.

[12] World Health Organization: Towards a Common Language for Functioning, Disability and Health: ICF – The International Classification of Functioning, Disability and Health. World Health Organization, 2002. www.who.int/classifications/icf/training/icfbeginnersguide.pdf (Accessed 19 April, 2013).

[13] Dmitrienko A, D'Agostino RB, Huque MF (2013). Key multiplicity issues in clinical drug development. Stat Med.

[14] Deeks JJ, Higgins JPT, Altman DG (Eds): Analysing data and undertaking meta-analyses. In: *Cochrane Handbook for Systematic Reviews of Interventions*, Version 5.1.0. Edited by Higgins JPT, Green S (Eds). The Cochrane Collaboration; 2011: Chapter 9.

[15] Sedgwick P (2013). What is a superiority trial?. BMJ.

[16] Sedgwick P (2013). Equivalence trials. BMJ.

[17] Sedgwick P (2013). What is a non-inferiority trial?. BMJ.

[18] Dent L, Raftery J (2011). Treatment success in pragmatic randomised controlled trials: a review of results funded by the UK Health Technology Assessment programme. Trials.

[19] Wellek S, Blettner M (2012). Establishing equivalence or non-inferiority in clinical trials. Dtsch Arztebl Int.

[20] Stovold E, Beecher D, Foxlee R, Noel-Storr A (2014). Study flow diagrams in Cochrane systematic review updates: an adapted PRISMA flow diagram. Syst Rev.

[21] Kiefer C, Rizzardi R, de Oliveira BF, Silva CM, Martins LP, Fantini CC (2012). Complexo homeopático na prevenção e tratamento de diarréias em leitões lactentes [Homeopathic complex in the prevention and treatment of diarrhoea in suckling piglets]. Rev Bras Saúde Prod Anim.

[22] Hoover TA: Epidemic diseases and homeopathic prophylaxis: fact or fiction. http://toddhoovermd.com/articles/epidemic-diseases-and-homeopathic-prophylaxis.html (Accessed, 26 June 2014).

[23] Ernst E, Lee MS (2008). A trial design that generates only ‘positive’ results. J Postgrad Med.

