# Supplementary material for: Veterinary homeopathy: Systematic review of medical conditions studied by randomised trials controlled by other than placebo
Source: BMC Vet Res. 2015 Sep 15;11:236. doi: 10.1186/s12917-015-0542-2 (PMC4570221; doi:10.1186/s12917-015-0542-2)
Supplement: Additional file 2: — PRISMA flowchart, illustrating records of RCTs that are eligible and ineligible for inclusion in the systematic review. * Records are numbered as per a previous paper published by the authors (Mathie et al., 2012) [1]. Papers presented in italics are those identified in the follow-up literature search. Excluded papers are shown in red. # RCT with continuous measure as main outcome (each unmarked trial has a dichotomous measure as the main outcome). (DOC 66 kb) [file 12917_2015_542_MOESM2_ESM.doc]

**Original search**: **20 records**

**OTP control**

* **2 RCTs**

**(Not treatment or prophylaxis)**:

A33. Sharma

A37. Trehan

**10 records**

**OTP control**

**(Treatment)**

**8 records**

**OTP control**

**(Prophylaxis)**

***Follow-up search****:*

***4 records***

***OTP control***

***(Treatment)***

*A39. Braun*

*A40. Lotfollahzadeh*

*A41. Catto*

*A42. Lohr*

* **2 RCTs (Combination intervention)**:

A29: Dreismann

A30: Lepple

* **14 RCTs**

**(Treatment)**

* **6 RCTs**

**(Prophylaxis)**

A12. Schütte

A13. Beceriklisoy

A14. Coelho

A15: Faulstich

A16. Klocke

A17. Rocha

A18. Sandoval #

A19. Silva #

A20. Varshney

A21. Zacharias #

*A39. Braun*

*A40. Lotfollahzadeh* #

*A41. Catto* #

*A42. Lohr*

A31. Reis #

A32. Reis #

A34. Signoretti #

A35. Sommer

A36. Soto

A38. Williamson

* **References**

A12 Schütte, A., 1988. Neue Ergebnisse aus Untersuchungen zur Behandlung des Puerperalsyndroms der Zuchtsauen [New results from investigations concerning the treatment of the puerperal syndrome of breeding sows]. *Der Praktische Tierarzt, Collegium Veterinarium* **19**, 67–73.

A13 Beceriklisoy, H.B., Özyurtlu, N., Kaya, D., Handler, J., Aslan, S., 2008. Effectiveness of Thuja occidentalis and Urtica urens in pseudopregnant bitches. *Wiener* *Tierärztliche Monatsschrift* **95**, 263–268.

A14 Coelho, C. de P., Soto, F.R.M., Vuaden, E.R., Melville, P.A., Oliveira, F.C.S., Benites, N.R., 2009. Evaluation of preventive homeopathic treatment against colibacillosis in swine production. *International Journal of High Dilution Research* **8**, 183–190.

A15 Faulstich, A., Lutz, H., Hellmann, K., 2006. Vergleich der Wirkung von Zeel® ad us. vet. bei durch nicht-infektiöse Gelenkentzündungen hervorgerufene Lahmheiten von Pferden mit Hyaluronsäure [Comparison of the effect of Zeel® ad us. vet. in lameness of horses caused by non-infectious arthropathy to the effect of hyaluronic acid]. *Der Praktische Tierarzt* **87**, 362–370.

A16 Klocke, P., Ivemeyer, S., Butler, G., Maeschli, A., Heil, F., 2010. A randomized controlled trial to compare the use of homeopathy and internal Teat Sealers for the prevention of mastitis in organically farmed dairy cows during the dry period and 100 days post-calving. *Homeopathy* **99**, 90–98.

A17 Rocha, R.A., Pacheco, R.D.L., Amarante, A.F.T., 2006. Efficacy of homeopathic treatment against natural infection of sheep by gastrointestinal nematodes. *Revista Brasileira de Parasitologia Veterinária* **15**, 23–27.

A18 Sandoval, C.H., Morfin, L.L., Lopez, B.B., 1998. Preliminary research for testing Baptisia tinctoria 30c effectiveness against salmonellosis in first and second quality broiler chickens. *British Homeopathic Journal* **87**, 131–134.

A19 Silva, N.L., Moletta, J.L., Minho, A.P., Filippsen, L.F., 2008. Use of biotherapic in the control of natural infestation by Boophilus microplus: pilot study. *International Journal of High Dilution Research* **7**, 35–37.

A20 Varshney, J.P., Naresh, R., 2005. Comparative efficacy of homeopathic and allopathic systems of medicine in the management of clinical mastitis of Indian dairy cows. *Homeopathy* **94**, 81–85.

A21 Zacharias, F., Guimarães, J.E., Araújo, R.R., Almeida, M.A.O., Ayres, M.C.C., Bavia, M.E., Mendonça-Lima, F.W., 2008. Effect of homeopathic medicines on helminth parasitism and resistance of Haemonchus contortus infected sheep. *Homeopathy* **97**, 145–151.

A29 Dreismann, G.M., 2010. Über die Wirkung eines homöopathischen Kombinationsarzneimittels auf stressabhängige Laborparameter beim Absetzen von Fohlen [Effects of the homeopathic preparation Engystol® ad us. vet. on stress induced parameters in weaning foals]. *Tierärztliche Umschau* **65**, 484–490.

A30 Lepple, F.J., 1984. Reduzierung der Endometritisgefahr bei Hündinnen nach Nidationsverhütung mit Östradiolbenzoat durch Sepia- und Pulsatillaextrakte [Reduction of endometritis risk in bitches after prevention of nidation with estradiol benzoate using extracts from Sepia and Pulsatilla]. *Kleintierpraxis* **29**, 203–208.

A31 Reis, L.S.L.S., Pardo, P.E., Oba, E., Kronka, S.N., Frazatti-Gallina, N.M., 2006. Matricaria chamomilla CH12 decreases handling stress in Nelore calves. *Journal of Veterinary Science* **7**, 189–192.

A32 Reis, L.S.L.S., Frazatti-Gallina, N.M., Lima Paoli, R., Giuffrida, R., Albas, A., Oba, E., Pardo, P.E., 2008. Efficiency of Matricaria chamomilla CH12 and number of doses of rabies vaccine on the humoral immune response in cattle. *Journal of Veterinary Science* **9**: 433–435.

A33 Sharma, M.L., Kansal, M.L., Ichhponani, J.S., 1987. Supplemental value of homoeopathic preparation on feed of commercial broilers. *Indian Journal of Poultry Science* **22**, 344–348.

A34 Signoretti, R.D., Veríssimo, C.J., De Souza, F.H.M., Garcia, T. da S., De Oliveira, E.M., De Souza, K.G., Mourão, G.B., 2008. Desempenho e infestação por parasitas em machos leiteiros suplementados com sal proteinado com ou sem os medicamentos homeopáticos [Performance and parasitologic infestation of male dairy cattle supplemented with proteic salt containing homeopathic medicines or not]. *Revista Brasileira de Parasitologia Veterinária* **17** (Suppl 1), 40–44.

A35 Sommer, H., Marx, D., 1972. Metaphylaktische Anwendung von Pulsatilla miniplex in der Sterilitätsbekämpfung des Rindes [Metaphylactic application of Pulsatilla miniplex in fertility control in cattle]. *Der Praktische Tierarzt* **53**, 45–46.

A36 Soto, F.R., Vuaden, E.R., Coelho, C. de P., Benites, N.R., Bonamin, L.V., de Azevedo, S.S., 2008. A randomized controlled trial of homeopathic treatment of weaned piglets in a commercial swine herd. *Homeopathy* **97**, 202–205.

A37 Trehan, P.K., 1994. Effect of some homoeopathic drugs on the performance in broilers. *Indian Journal of Animal Research* **28**, 23–26.

A38 Williamson, A.V., Mackie, W.L., Crawford, W.J., Rennie B., 1991. A study using Sepia 200c given prophylactically postpartum to prevent anoestrus problems in the dairy cow*. British Homeopathic Journal* **80**, 149–156.

*A39 Braun, G., Hellmann, K., Reinhart, E., 2011. Behandlung von puerperaler Septikämie und Toxämie (MMA) bei Zuchtsauen mit biologischer Therapie im Vergleich zu einer Standardbehandlung [Treatment of puerperal septicaemia and toxaemia (MMA) with biological therapy in sows compared to a Standard therapy].* Tierärztliche Umschau *66,* *303–311.*

*A40 Lotfollahzadeh, S., Alizadeh, M.R., Mohri, M., Dezfouli, M.R.M., 2012. The therapeutic effect of Tarentula cubensis extract (Theranekron (R)) in foot-and-mouth disease in cattle: a randomised trial in an endemic setting.* Homeopathy ***101****, 159–164.*

*A41 Catto, J.B., Bianchin, I., Feijó, G.L.D., Araújo, F.R., Ramos, C.A. do N., Castelão, A.B.C., 2013. Weight gain and control of endo- and ectoparasites of Beef heifers treated with allopathic, herbal and homeopathic drugs.* Revista Brasileira de Parasitologia Veterinária ***22****, 502–510.*

*A42 Lohr, B., Braun, G., Gasda, N., Hellmann, K., Reinhart, E., 2012. Treatment of neonatal diarrhoea in calves with natural medicine compared to a standard therapy.* Der Praktische Tierarzt ***93****, 150–158.*
